# Molecular insight into optimizing the N- and P-doped fullerenes for urea removal in wearable artificial kidneys

**DOI:** 10.1007/s10856-021-06525-7

**Published:** 2021-04-23

**Authors:** Ahmad Miri Jahromi, Pegah Zandi, Mohammad Khedri, Ebrahim Ghasemy, Reza Maleki, Lobat Tayebi

**Affiliations:** 1 Computational Biology and Chemistry Group (CBCG), Universal Scientific Education and Research Network (USERN), Tehran, Iran; 2 School of Metallurgy and Materials Engineering, College of Engineering, University of Tehran, Tehran, Iran; 3 Nanotechnology Department, School of New Technologies, Iran University of Science and Technology, Tehran, Iran; 4 Marquette University School of Dentistry, Milwaukee, WI 53233 USA

## Abstract

Urea is the result of the breakdown of proteins in the liver, the excess of which circulates in the blood and is adsorbed by the kidneys. However, in the case of kidney diseases, some products, specifically urea, cannot be removed from the blood by the kidneys and causes serious health problems. The end-stage renal disease (ESRD) patients are not able to purify their blood, which endangers their life. ESRD patients require dialysis, a costly and difficult method of urea removal from the blood. Wearable artificial kidneys (WAKs) are consequently designed to remove the waste from blood. Regarding the great amount of daily urea production in the body, WAKs should contain strong and selective urea adsorbents. Fullerenes—which possess fascinating chemical properties—have been considered herein to develop novel urea removal adsorbents. Molecular dynamics (MD) has enabled researchers to study the interaction of different materials and can pave the way toward facilitating the development of wearable devices. In this study, urea adsorption by N-doped fullerenes and P-doped fullerenes were assessed through MD simulations. The urea adsorption was simulated by five samples of fullerenes, with phosphorous and different nitrogen dopant contents. For comparing the urea adsorption capacity in the performed simulations, detailed characteristics—including the energy analysis, radius of gyration, radial distribution function (RDF), root-mean-square fluctuation (RMSD), and H-bond analyses were investigated. It had been determined that the fullerene containing 8% nitrogen—with the highest reduction in the radius of gyration, the maximum RDF, a high adsorption energy, and a high number of hydrogen bonds—adsorbs urea more efficiently.

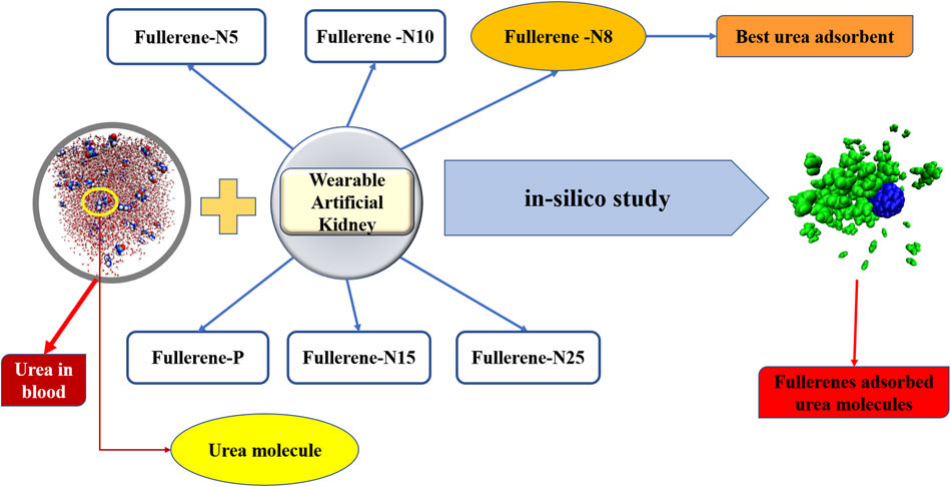

## Introduction

The breakdown of the proteins in the liver results in the formation of waste products such as urea. The excess urea that is formed is adsorbed from the blood by kidney and consequently removed by urine. In chronic kidney disease, the patient may experience the complete destruction of their kidneys. End-stage renal disease (ESRD) refers to a condition in which kidney activity is reduced to <10% [1], and 50–75% of the nephrons lose their function, making the ESRD patients in urgent need for kidney transplantation [2–5]. The skeletal, endocrine, cardiovascular, hematological, and gastric systems are all affected by kidney failure, endangering the lives of ESRD patients [6]. The ESRD patients also require dialysis, which is an artificial blood purification process. This process has several drawbacks, including the high cost and disturbance of the patients' normal lives [7–9] . Employing wearable artificial kidney (WAK) devices is an emerging approach. WAKs are advantageous due to the elimination of the clinical chair time requirement in the conventional dialysis, as the WAKs can purify blood 168 h per week, and as a result ESRD patients can carry WAKs and continue their normal life [10–14].

The WAKs can purify the creatinine, urea, phosphorus, and other bodily wastes. Therefore, these devices are good alternatives for the dialysis process. One of the main limitations of WAK devices is their urea removal capacities due to the high volume of urea production. Besides, these devices do not carry high enough volumes of dialysis fluid to make them light weight and portable.

Currently, the most effective way to get rid of urea is to use special enzymes to hydrolyze the urea in an aqueous solution. In this method, according to Reaction 1, the urea is converted to ammonium and ammonium bicarbonate in the presence of water. The ammonium produced is more dangerous than the urea, so the devices are designed to absorb the ammonium products by using engineered materials such as zirconium. Although this method has a considerable efficiency for urea adsorption, the great amount of zirconium phosphate which is needed to absorb the produced ammonium makes it bulky and heavy. On the other hand, the decomposition of ammonium bicarbonate into carbon dioxide and water, which necessitates the elimination of carbon dioxide, makes zirconium less efficient [14–17]. As a result, many studies have been done regarding urea removal.

**Fig. 1:**
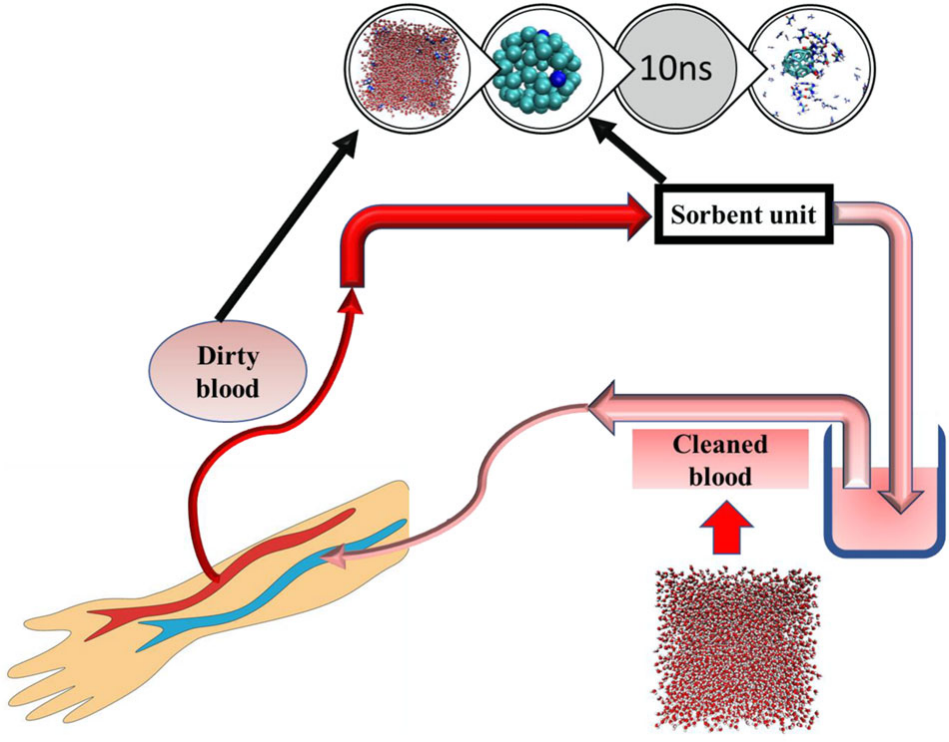
Schematic representation of the blood urea purification by the adsorbents used in the WAK devices. Dirty blood in the blue vein pumps into the adsorbent part of the WAK to remove urea, and then the clean blood pumps back into the red vein

Adsorbents are another outstanding method in this regard. The adsorption of urea by adsorbents does not bring about the problems related to the production of toxic by-products, but instead, attempts to eliminate them. Hence, the WAK devices become smaller and lighter. Therefore, many studies have been done to discover the efficient urea adsorbents. These adsorbents include activated carbon [18–22], silica [22, 23], zeolites [18, 23], chitosan [24–27], and MXenes [28, 29]. However, all of the used adsorbents had a low adsorption affinity and capacity, such that they could not be effective in eliminating the high urea content in the urine. Figure 1 demonstrates a very simple extraction of excess blood urea by an adsorbent used in a WAK device.

1 $${\mathrm{CN}}_2{\mathrm{H}}_4{\mathrm{O}} + 2{\mathrm{H}}_2{\mathrm{O}}\, + \,{\mathrm{H}}^ + \mathop{\longrightarrow}\limits^{{{\mathrm{urease}}}}{\mathrm{CO}}_3{\mathrm{H}}^ - + 2{\mathrm{NH}}_4^ +$$

In this paper, the N- or P-doped fullerenes are studied for the first time to remove the urea present in the blood. As the Wang et al. [30] theoretically explained, N-doped fullerenes are the best choice for facilitating the oxygen reduction reaction process, and similarly it may affect the adsorption of urea. Thus, in order to compare the efficiency, doping different concentration of nitrogen has been simulated in this study. Moreover, P-doping as Khan et al. [31] claimed can affect the removal of Co_2_, N_2_, and products beside urea. Nitrogen and phosphorus atoms replace a number of carbon atoms. The effect of different amounts of nitrogen on the structure of the fullerenes has also been investigated and has been compared to a certain amount of phosphorous. This enables us to clearly compare the effect of two different dopants (P, N) to see which form of urea removal has better performance. The purpose of the simulations is to investigate the urea adsorption by the pristine fullerene, and also the fullerene structures prepared by incorporating the dopants. The doped fullerene species possess highly effective physical and chemical properties.

Due to the fact that investigating the adsorption behavior is time consuming and expensive in the laboratory, molecular dynamics (MD) simulation—a powerful tool with very high accuracy—can be considered, which has extensive applications in various fields [32–34]. MD simulate the atomic and molecular motions during a defined period. The MD simulations can be employed for the thermodynamic study of a material’s behavior in three phases—namely liquid, gas, and solid—using the velocity, location, and energy of the particles.

To this end, the urea adsorption by N- and P-doped fullerene is compared for structures containing various dopant contents. The urea adsorption by pristine fullerene, fullerene-N*X* (*X* = 5, 8, 10, 15, 25, denoting the percentage of carbon atoms replaced by nitrogen atoms), and fullerene-P (phosphorus atoms replaced 50 percentage of carbon atoms of fullerenes) are studied and compared. To the best of our knowledge, this is the first-time consideration of N- or P-doped fullerene nano-sorbents being used in WAKs, which can pave the way toward experimental works and facilitating the improvement of a human’s living quality. For this purpose, detailed methods are employed to assess the RMSD, radius of gyration, RDF, and H-bond and energy analyses, which are all essential in evaluating an adsorbent.

## Material and methods

### Molecular dynamics simulation

In the MD simulation, interatomic interactions can be examined by the potential energy of each atom. Fixed time intervals are considered for the numerical calculation of interactions. In the following relations, “re” shows the distance between the two atoms, while *σ* indicates the depth of the potential well, and *V* denotes the potential between the two atoms. The potential energy can be calculated from Eqs. (2)–(5) [35–37]:

2 $${U} = {U}\,\left( {{\mathrm{re}}} \right)$$

3 $${Fi} = - {dv}/{dri}$$

4 $${mi}\,\left( {d2{ri}/{dt}2} \right) = {Fi},\,{i} = 1,...,{N}$$

5 $${V}_{{\mathrm{vdW}}} = 4\varepsilon \,\left[ {\left( {\sigma /{\mathrm{re}}} \right)^{12} - \left( {\sigma /{\mathrm{re}}} \right)^6} \right]$$

### Simulation method

The structure of fullerenes was made by the Nanotube_Modeler_1.7.9 software [38, 39]. Then, using the Avogadro software, some of its carbon atoms were replaced by nitrogen and phosphorus. The N-doped and P-doped fullerene structures were optimized by the Gaussian 09 [40–42] software. Optimized potentials for liquid simulations force field had been used for designing all of the molecular structures [43, 44]. The size of the simulation box was set at 6 nm, which included 6550 water molecules and 80 urea, as well as one fullerene ball, and each simulation box contains one fullerene ball/adsorbent particle. After providing the required files, four simulation steps were performed. Optimization was conducted for 50,000 1-fs time steps. The force field is the most important factor in the MD processes [45]. The system size in the MD simulations should be very small, just a few nanometers. The behavior of the system can be determined by calculating the interactions between the constituents. The complex processes of the living systems can be investigated by MD, for instance, the adsorption of nanoparticles or the acting mechanism of the drugs. The information provided by the MD simulation is at the microscopic level (i.e., atomic speed and position). These microscopic properties can be then related to the macroscopic features (e.g., energy, pressure, and specific heat) through statistical mechanics. Statistical systems with a large number of components (such as atoms, molecules or fundamental particles) can be analyzed by the statistical mechanics.

The energy level was set to 100 kJ/mol, and in the NVT stage, the simulation box temperature was balanced at 300 K using the v-rescale algorithm [46]. The NVT simulation time was 100 ps. In the NPT stage, the pressure was balanced at 1 bar using the Parrinello–Rahman algorithm in 100 ps [47]. Finally, the simulation was performed by the linear constraint solver algorithm, taking into account the H-bond and the cut-off radius of 1.4 nm in 10 ns [48].

## Results

At the beginning of the simulation, the urea molecules were irregularly placed in the simulation box. The positioning of the urea molecules next to the fullerenes was affected by formation of the intermolecular forces. The force generated between the urea molecules and fullerenes causes the urea molecules to move in the simulation box. The strong adsorption of urea causes its sticking to the studied fullerene surface. The position of the molecules can be seen in the final images of the simulation. Therefore, the use of simulation images are effective tools for monitoring the adsorption of urea by fullerenes. Using the visual molecular dynamics [49] software, the initial and final images of the simulation are taken.

**Fig. 2:**
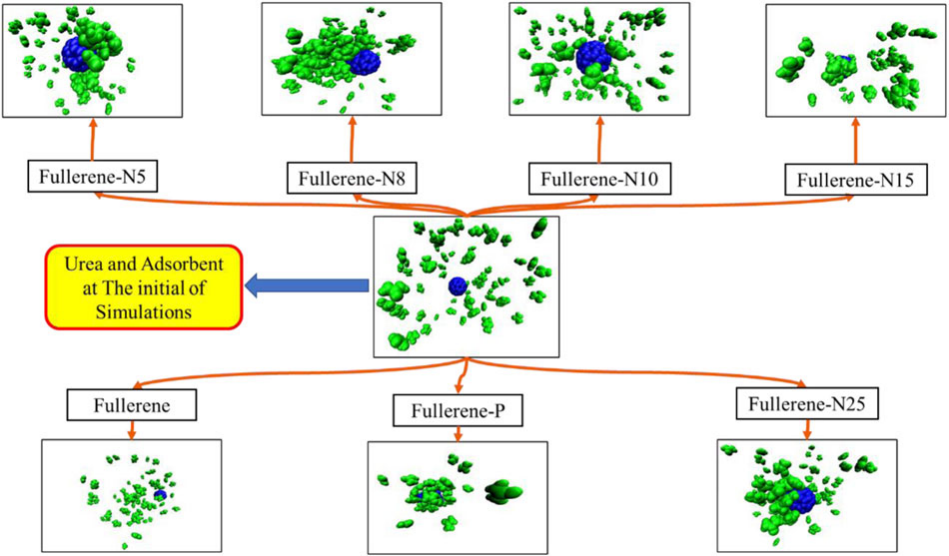
Urea adsorption by different type of fullerenes after 10 ns (blue and green particles refers to the fullerene the urea molecules, respectively)

Figure 2 shows the position of urea molecules relative to the Fullerene-N5, Fullerene-N8, Fullerene-N10, Fullerene-N15, Fullerene-N25, Fullerene-P, and the pristine fullerene at the beginning and end of the simulation. As seen, urea molecules accumulate around all adsorbents at the end of the simulation, reflecting the urea adsorption by all adsorbents. However, simulation images alone are not sufficient for comparing the urea adsorption affinity of the modeled sorbents. Therefore, the analyses like, RMSD, number of H-bonds, radius of gyration, RDF, and energy analyses are exploited to investigate the urea adsorption intensity by nanoparticle simulations.

### The Gibbs free energy of simulations

The umbrella analysis was used to calculate the change in Gibbs free energy. To perform the umbrella analysis, we separated one of the adsorbed urea molecules by using the pull code. Then, 100 configurations were extracted from the obtained frames, and the simulation was carried out by using the Berendsen pressure and temperature algorithms in 10 ns with a time step of 2 fs. Next, using the WHAM analysis, the Gibbs free energy of the urea adsorption by the modeled structures were calculated. Gibbs free energy, considering the entropy and enthalpy of urea adsorption, is the most important indicator for evaluating the stability of the adsorption simulations by different adsorbates. Table 1 shows the Gibbs energy values for the urea adsorption simulations by the doped fullerene structures. The negative values of Gibbs free energy show that the fullerene-based sorbents were able to adsorb the urea molecules well.

**Table 1:** The Gibbs free energy of the urea adsorption simulations on fullerene structures

Adsorbent	Fullerene-N5	Fullerene-N8	Fullerene-N10	Fullerene-N15	Fullerene-N25	Fullerene-P	Fullerene
Gibbs free energy(kcal/mol)	−15.76	−22.49	−20.17	−10.62	−8.48	−17.93	−7.04

### The root mean square deviation (RMSD) analysis of the nano systems

**Fig. 3:**
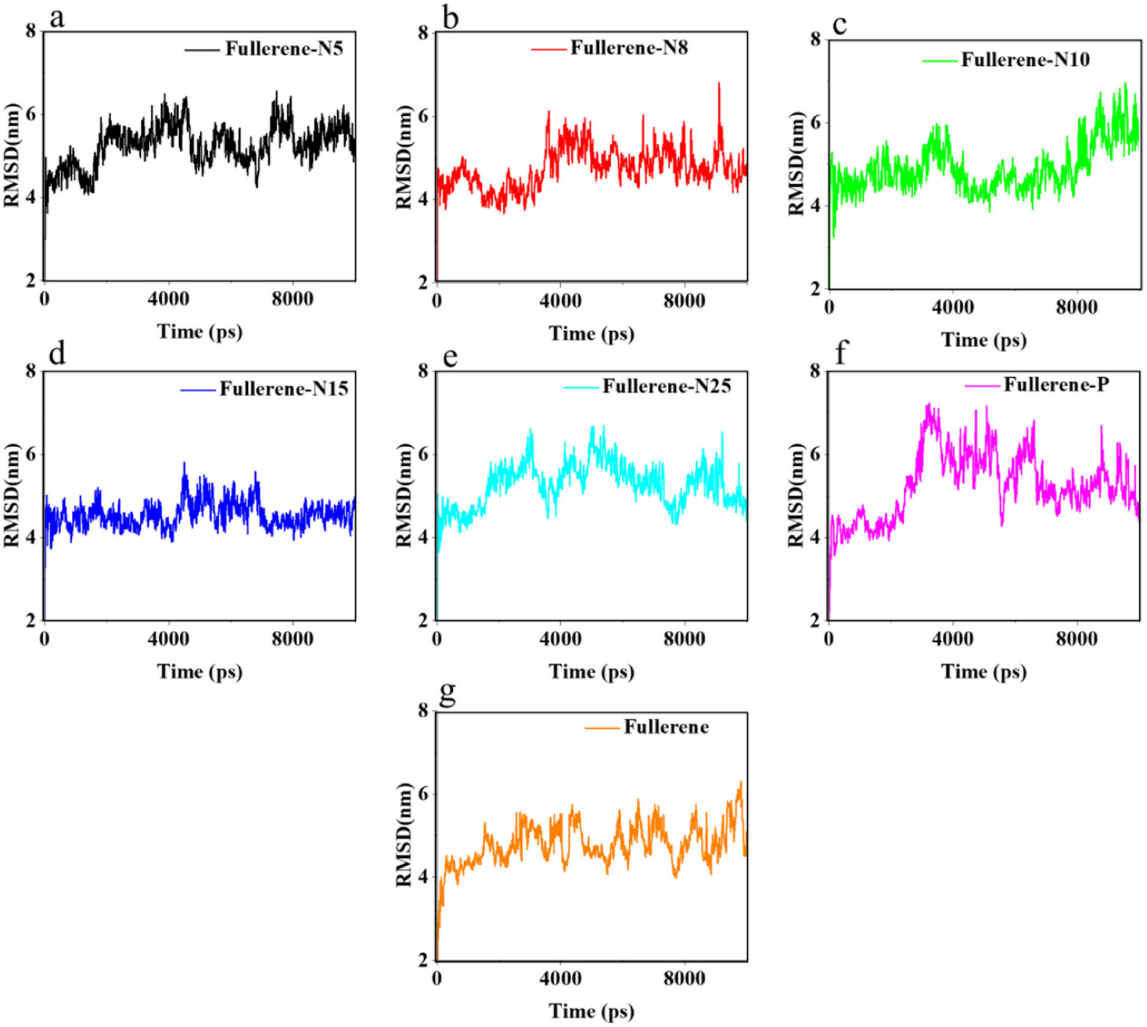
RMSD graph of (**a**) Fullerene-N5, (**b**) Fullerene-N8, (**c**) Fullerene-N10, (**d**) Fullerene-N15, (**e**) Fullerene-N25, (**f**) Fullerene-P, and (**g**) Fullerene simulation during the simulation course

Figure 3 shows the RMSD values for the considered structures. In the process of urea adsorption by fullerenes, urea molecules are transported to be located at the level of fullerenes. The stability of urea and the named fullerenes make it possible to eliminate the excess urea in the body. In the MD simulation, the stability is calculated via the root mean square deviation (RMSD) analysis. The RMSD estimation goes as below:

6 $${\rm{RMSD}} = \sqrt {\frac{1}{N}\mathop {\sum}\nolimits_{j = 1}^N {\left( {Cj\left( t \right) - Cr\left( t \right)} \right)^2} }$$

where *N* is number of atoms, *Cj*(*t*) is coordinate of atom *j* at time *t*, and *Cr*(*t*) is the coordinate of the reference atom at time *t*. In the RMSD analysis, the oscillations of the molecules are calculated relative to the desired reference. Greater fluctuations represent the more unstable simulation systems [50–53]. Figure 3 shows the RMSD diagrams for Fullerene-N5, Fullerene-N8, Fullerene-N10, Fullerene-N15, Fullerene-N25, Fullerene-P, and Fullerene structures.

### Hydrogen interactions of urea and fullerenes

In some hydrogen compounds, there is a strong intermolecular attraction due to the presence of hydrogen bonds (H-bond). H-bond are formed between hydrogen atoms and small-size elements with high electronegativities. This type of bonds can be formed not only between the molecules of the same substance, but also between two different molecules. One H atom and the pair of non-common electron molecules participate to form a H-bond [54–56].

The H-bond created during the simulation is an effective factor for the adsorption of urea molecules at the surface of the adsorbent. Strong interactions of the simulated molecules are the result of the number of H-bond. This can be a good indicator of the intensity of urea adsorption in different simulators. The H-bond analysis calculates the number of H-bond between the simulated molecules. H-bond are non-binding interactions occurring as a hydrogen atom bonds to an electron donor and an electron acceptor through electrostatic interactions. Also, the average number of H-bond between urea and fullerene systems during 10,000 ps of MD simulation are reported. Table 2 represents the average number of H-bond which lead to quite fixed ligands binding. Moreover, this paper discusses the number of H-bond as an indicator to compare the urea adsorption of the studied adsorbents. The urea molecule is composed of amine functional groups, which are capable of forming H-bond. As it is shown in Table 2, the greatest value for the average number of H-bonds belongs to Fulllerene-N25, while Fullerene-P and Fullerene seem not to have any H-bond.

**Table 2:** The average number of hydrogen bonds between urea and mentioned fullerene systems during 10,000 ps of MD simulation

Adsorbent	Fullerene-N5	Fullerene-N8	Fullerene-N10	Fullerene-N15	Fullerene-N25	Fullerene-P	Fullerene
Average of hydrogen bonding	0.136	0.369	0.439	0.583	0.778	0	0

### Intermolecular bonds by energy analysis

Placing urea and fullerene molecules in the simulation box and allowing intermolecular interactions between the two substances causes intermolecular bonds. Electrostatic, hydrogen, and van der Waals (vdW) forces are types of bonds resulting from the intermolecular interactions. The higher the number of bonds formed, the greater the intensity of urea adsorption in the simulation. As the intermolecular interactions increase, the absolute value of the simulation energy enhances. Therefore, comparing the energy resulting from intermolecular bonds during the simulation (Table 3) is a good criterion for comparing the adsorption intensity of urea molecules by different adsorbents.

**Table 3:** The average vdW, electrostatic, and total energy of the studied systems and urea adsorption

Adsorbent	Fullerene-N5	Fullerene-N8	Fullerene-N10	Fullerene-N15	Fullerene-N25	Fullerene-P	Fullerene	Ti_3_C_2_OH [29]
Total energy (kJ/mol)	−76.687	−170.875	−100.612	−69.001	−56.015	−98.123	−37.923	83.4598
van der Waals Energy (kJ/mol)	−76.352	−170.013	−100.162	−68.723	−55.771	−97.748	−37.811	
Electrostatic energy (kJ/mol)	−0.3346	−0.8618	−0.449	−0.277	−0.243	−0.375	−0.112	

In the MD simulation of energy analysis, the energy generated by vdW and electrostatic bonds is calculated. The energies from the electrostatic bonds and vdW bonds in the simulations for Fullerene-N5, Fullerene-N8, Fullerene-N10, Fullerene-N15, Fullerene-N25, Fullerene-P, and Fullerene are calculated by the mmpbsa [57, 58] software. The sum of the energies resulting from the intermolecular bonds is a good indicator for the intensity of urea adsorption in the simulations performed in this paper [59–61].

As Table 3 indicates, Fullerene-N8 possesses the highest average value for both vdW and electrostatic energy, along with the total energy of −170.875 kJ/mol. On the other hand, the minimum value for energy value belongs to fullerene with an average value of −37.923 for the total energy. Compared to the Ti_3_C_2_OH, which is an efficient MXene developed for WAK [29], the fullerene-N8 is predicted to be better.

### The radius of gyration analysis of the nano systems

**Fig. 4:**
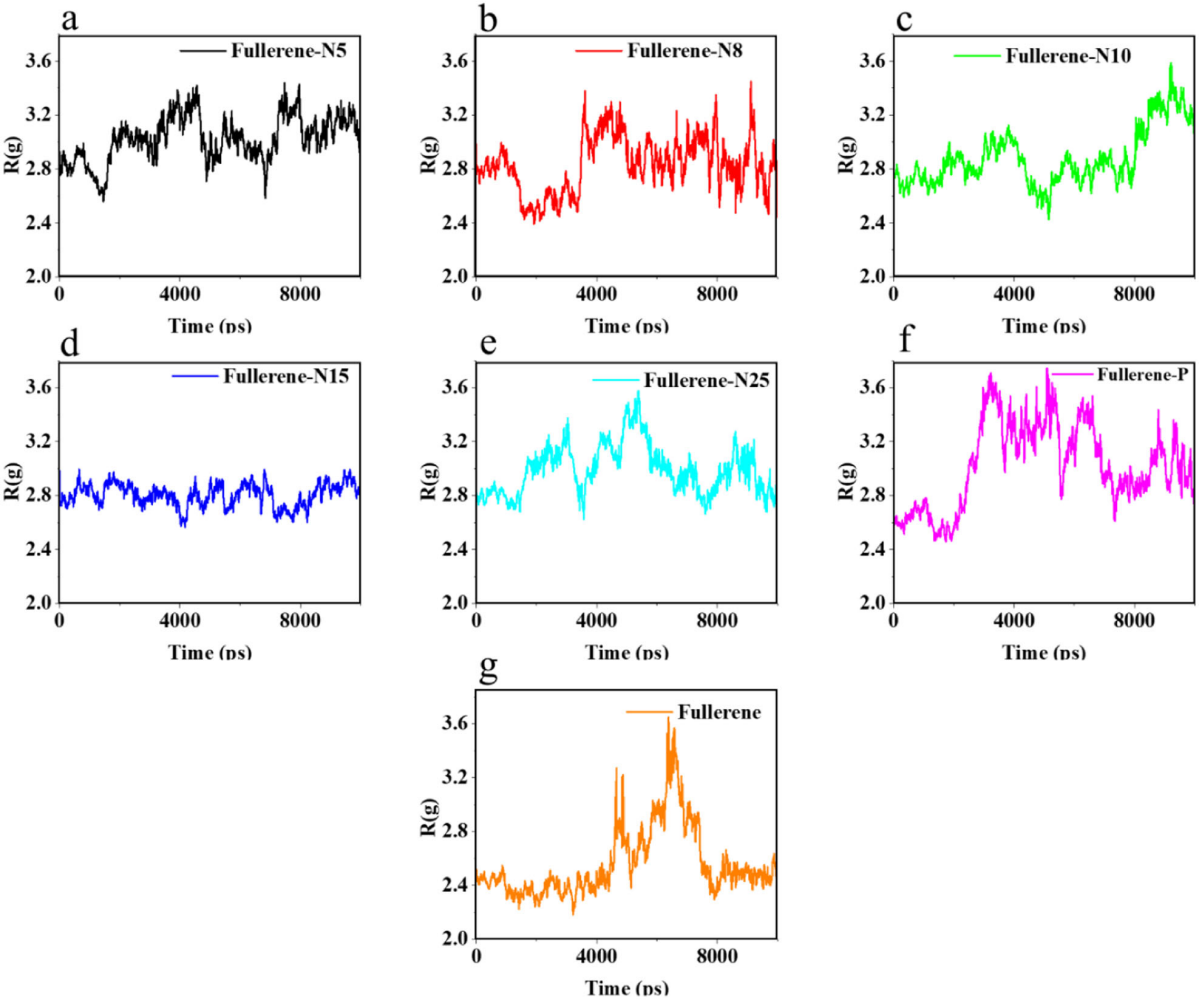
Radius of gyration in (**a**) Fullerene-N5, (**b**) Fullerene-N8, (**c**) Fullerene-N10, (**d**) Fullerene-N15, (**e**) Fullerene-N25, (**f**) Fullerene-P, and (**g**) Fullerene simulations during 10 ns

The higher the urea adsorption rate in the simulation, the higher the compression of the urea molecules (Fig. 4). Radius of gyration is calculated as below:

$$R_g^2 = \frac{1}{M}{\sum} {\left( {m_{ia}\left( {\overrightarrow r _{ia} - \overrightarrow r _{cm,i}} \right)} \right.}$$

where *M* is the molecular weight of the molecule, *m*_*ia*_ and $\overrightarrow r _{ia}$ are the weight and position of atom *a* in molecule i, and $\overrightarrow r _{cm,i}$ is the center of mass of molecule *i* [62]. Analysis of the radius of gyration shows the accumulation of particles at different simulation times. As a result, using the radius of gyration analysis, the urea compression created during the simulation can be investigated. Reducing the final radius of gyration indicates more particle compression. Therefore, the analysis of the radius of gyration is effective for urea adsorption, as well as a comparison between its first and final value for each structure [63–65].

Table 4 shows the first 10 ns of the *R*(*g*) simulation for the investigated structures. Combining the results from Fig. 4 and Table 4, it can be illustrated that the highest and the lowest *R*(*g*) values belong to fullerene-N8 and fullerene, respectively.

**Table 4:** The difference between the initial and final grating radii in the urea adsorption simulations in the presence of different types of fullerenes

Adsorbent	Fullerene-N5	Fullerene-N8	Fullerene-N10	Fullerene-N15	Fullerene-N25	Fullerene-P	Fullerene
*Rg*(*t* = 0) − *Rg*(*t* = 10 ns)	0.236	0.54	0.393	0.189	0.102	0.308	0.037

### The radial distribution function (RDF) analysis

The gravity created between urea molecules and adsorbents changes the urea density at different points in the simulation box. The surface adsorption of urea molecules maximizes the urea density at a close proximity to the absorbent materials. In the MD simulation, particle trajectory can be calculated using the RDF analysis. The RDF analysis shows the accumulation of urea molecules in terms of distance from a tagged particle. A higher maximum RDF value indicates more urea adsorption. The RDF analysis is the best indicators for comparing the urea adsorption intensity of the simulated nanoparticles. In this paper, the RDF analysis is performed between urea and Fullerene-N5, Fullerene-N8, Fullerene-N10, Fullerene-N15, Fullerene-N25, Fullerene-P, and Fullerene adsorbents. The horizontal axis shows the distance of different particles from a tagged particle. Moreover, each peak broadening represents a particle’s vibration and the density of the surrounding matter varies as a function of the distance from a tagged particle [66].

**Fig. 5:**
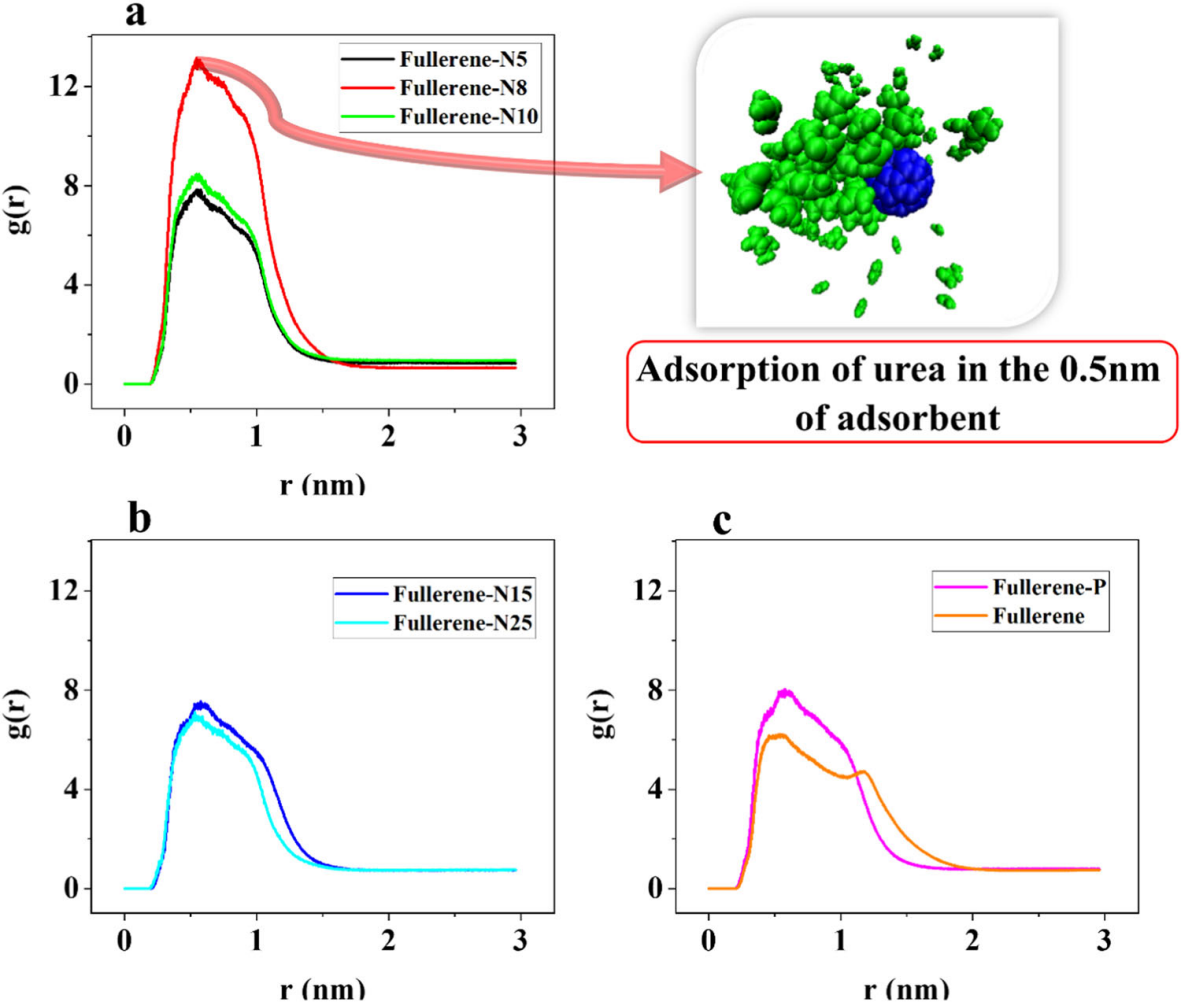
RDF diagrams of (**a**) Fullerene-N5, N8, and N10, (**b**) N15, N25, and (**c**) Fullerene and Fullerene-P systems

Figure 5a shows the RDF diagrams of the Fullerene-N5, Fullerene-N8, and Fullerene-N10, in which Fullerene-N8 shows the most intense peak in a more near distance.

Figure 5b shows the RDF diagrams of Fullerene-N15 and Fullerene-N25. The maximum RDF values of the Fullerene-N15 and Fullerene-N25 simulations are 7.532 and 7.109, respectively.

Figure 5c shows the RDF diagram of the Fullerene and Fullerene-P simulations. The maximum RDF of the Fullerene and Fullerene-P simulations emerges at 6.219 and 8.041, respectively. Moreover, Table 5 summarizes the maximum RDF for all of the abovementioned structures. As it can be indicated from Table 5, the maximum peak occurs in the fullerene-N8 simulation, with its value of 13.136 being the highest, while the peak for fullerene with a value of 6.219 is the lowest value among the investigated structures.

**Table 5:** Maximum values of the RDF diagram simulation of urea adsorption in the presence of different adsorbents

Adsorbent	Fullerene-N5	Fullerene-N8	Fullerene-N10	Fullerene-N15	Fullerene-N25	Fullerene-P	Fullerene
Max of RDF	7.843	13.136	8.462	7.532	7.109	8.041	6.219

## Discussion

In order to design a light and high-quality WAK, which is capable to be used frequently and efficiently in a patient’s body, we did a simulation to investigate the probability of using N*X*-doped (*X* = 5, 8, 10, 15, 25) fullerene (due to the theorically explained adsorption behavior) and P-fullerene and compared their capacities with the bare fullerene. So, along with observing the simulation images, different analysis methods were employed to qualify the investigated materials to purify the blood. These results were indeed substantial in proposing the N- and P-doped fullerenes as efficient nanostructures for urea removal. In this regard, the negative Gibbs free energy values indicated the relative stability of the modeled systems, which stands for the suitability of the proposed adsorbents to remove urea. The Gibbs free energy value of the urea adsorption simulations in the presence of N-doped and P-doped fullerene compared to that of the pristine fullerene, demonstrated the positive effect of nitrogen and phosphorus dopants on the urea adsorption. In this regard, fullerene-N8 with the Gibbs free energy value of −33.49 kJ/mol shows the best performance. In addition to the variation of Gibbs free energy, the RMSD analysis was regarded to peruse the modeled systems’ stability. In accordance with the energy analysis, the RMSD investigation indicated better stability of the systems comprising of the doped fullerene samples and urea molecules, compared to that of the pristine fullerene. The RMSD simulation of Fullerene-N8 in the range of 1–3 ns indicated the relative stability of the simulation system after the optimization step. The RMSD simulation of the Fullerene-N8 has longer time ranges with a slope of zero, which represents its higher stability.

It was also revealed that the fullerene samples, which possessed nitrogen, could establish hydrogen bonding with urea molecules which could contribute to the better interactions between N-doped fullerene and urea contaminants. As a result, the higher the numbers of N-doping become, the better adsorption it provides. Although, the N25 has a better interaction based on the number of H-bond it results in, in order to decide on the best option for WAK, we should also consider the compactness, flexibility, and other effective parameters. In this regard, the radius of gyration diagrams illustrated that the fullerene samples which possessed nitrogen or phosphorus dopants could provide a greater difference in radius of gyration. This is attributed to the stronger compression of the urea molecules around the doped fullerene models and a better adsorptive affinity. The differences in the calculated radius of gyration indicate the positive effect of substituting phosphorus and nitrogen atoms on the molecular structure of fullerenes. Newly built structures could further compress the urea molecules. However, the greatest reduction in the radius of gyration, and consequently, the highest compression of the urea molecules occurs in the Fullerene-N8 simulation.

The energy analysis clarified that introducing the N and P dopants into the fullerene structure brought about a significant effect on the vdW bonds. Moreover, the polarity of the fullerene was ameliorated, and accordingly stronger interactions were established. In addition to elucidating the effect of N dopants on improving the adsorption energy, the optimum amount of the N dopants was also determined, which can be considered in optimizing the fullerene adsorbates for urea removal. The average vdW energy of the Fullerene-N8 and Fullerene-P were −170.875 and −97.748 kJ/mol, respectively. The greater average absolute value of the vdW energy in Fullerene-N8 of −170.013 kJ/mol implied more vdW bonds and, thus, a greater urea adsorption. According to the RDF analysis, it was revealed that the N-doped fullerene structures possessed a higher maximum RDF than the pristine fullerene. This is another index attesting the effective role of N and P-doping in modifying the adsorptive affinity of fullerene toward the urea removal. In the Fullerene-N8, the highest maximum RDF and the stronger interactions between urea resulted in a higher urea density, as well a greater urea adsorption.

The simulations clearly confirmed that doping nitrogen and phosphorous into the fullerene structure can effectively modify its adsorption performance. Moreover, in addition to investigating the role of dopants (N and P) on the adsorption capacity of the fullerene, the best amount of doping among the studied dopants has been found. It was confirmed that N-doping can induce hydrogen bonding, improve the adsorption energy, and ameliorate the adsorption stability. In addition, the simulations demonstrated that there is an optimum nitrogen content which can lead to the best performance, less or more than which can deteriorate the adsorption performance.

This novel work attests to the successful application of N8-doped fullerene in urea removal, and suggests that it can be employed in the WAK devices. In addition to high adsorption capacity, high tunability, and good biocompatibility, N-doped fullerenes also have a low production cost, so they could be attractive options for further studies on the removal of urea and other kidney toxins. In this study, for the first time, molecular simulations and mechanistic studies have been used to investigate the removal of urea in the WAK devices. Mechanistic studies can provide a detailed and in-depth insight into the phenomenon of urea adsorption. In fact, with atomistic simulations, adsorbents can be adjusted and engineered according to accurate atomic analysis. For the future works, we suggest the in vitro and in vivo studies on the N-doped fullerene nano-sorbents to facilitate the development of wearable devices to deploy the emerging technologies in humans’ well-being.

## Conclusion

In this paper, the urea adsorption by fullerene and N- and P-doped fullerene is investigated through MD simulations. The results reveal that although the fullerene itself cannot be a suitable option for urea removal, the phosphorus and nitrogen dopants could improve the stability of the urea adsorption by fullerene structure. It is found that the nitrogen-containing fullerene sorbents, in contrast to the P-doped and pristine fullerene, could establish H-bond with urea molecules, which are effective in capturing the urea. Moreover, the energy analysis—as an important index of interactions—demonstrates that both N and P dopants could enhance the adsorption energy significantly. The optimum amount of nitrogen content is also determined through the energy analyses, which have a much better performance compared to the P-doped fullerene. The radius of gyration and RDF analysis both are in line with the energy analysis, which attest to the success of N- and P-doping in ameliorating the adsorption affinity of the fullerene. The presented results clearly demonstrate that the N-doped fullerenes, especially N8, are the most suitable studied adsorbent for urea removal and a simple increase in the number of N-doping does not necessarily enhance the fullerene absorbance. Thus, the N8-fullerene can be tailor-made in future experiments and will help in utilizing the wearable devices at the service of human health.
